# Protein kinase C theta is dispensable for suppression mediated by CD25^+^CD4^+^ regulatory T cells

**DOI:** 10.1371/journal.pone.0175463

**Published:** 2017-05-22

**Authors:** Kerstin Siegmund, Nikolaus Thuille, Katarzyna Wachowicz, Natascha Hermann-Kleiter, Gottfried Baier

**Affiliations:** Department for Pharmacology and Genetics, Medical University Innsbruck, Innsbruck, Austria; University Medical Center of the Johannes Gutenberg University of Mainz, GERMANY

## Abstract

The activation of conventional T cells upon T cell receptor stimulation critically depends on protein kinase C theta (PKCθ). However, its role in regulatory T (Treg) cell function has yet to be fully elucidated. Using siRNA or the potent and PKC family-selective pharmacological inhibitor AEB071, we could show that murine Treg-mediated suppression *in vitro* is independent of PKCθ function. Likewise, Treg cells of *PKCθ*-deficient mice were fully functional, showing a similar suppressive activity as wild-type CD25^+^CD4^+^ T cells in an *in vitro* suppression assay. Furthermore, *in vitro*-differentiated wild-type and *PKCθ*-deficient iTreg cells showed comparable Foxp3 expression as well as suppressive activity. However, we observed a reduced percentage of Foxp3^+^CD25^+^ CD4^+^ T cells in the lymphatic organs of *PKCθ-*deficient mice. Taken together, our results suggest that while PKCθ is involved in Treg cell differentiation *in vivo*, it is dispensable for Treg-mediated suppression.

## Introduction

Regulatory T (Treg) cells are important for maintaining immune homeostasis and self-tolerance. Their critical role in immune regulation is illustrated by an overt autoimmune phenotype observed in mice and humans, which lack functional Treg cells due to mutations of the key Treg-defining transcription factor Foxp3 ([1, 2]; for an overview see Ramsdell and Ziegler [3]). Several groups have shown that the thymic development of CD25^+^CD4^+^ Treg cells essentially depends on intact T cell receptor (TCR) signaling ([4] and references therein). In particular, high-affinity antigens that generate strong TCR signals drive the commitment of developing thymocytes into the Treg lineage [5–11]. Moreover, tonic TCR signaling has been suggested to be important for the maintenance of the Treg signature and peripheral homeostasis [12–14]. In addition, TCR stimulation appears to be vital for the suppressive activity of Treg cells [15, 16]. Indeed, Treg cells deficient in proximal TCR signaling molecules such as Lck [12], LAT [17, 18] or the TCRα chain itself [13, 14] exhibit impaired suppressive activity. Furthermore, the inhibition of negative regulators of TCR signaling such as the phosphatase SHP-1 or the diacylglycerol (DAG)-metabolizing kinase DGKζ [19, 20] results in an augmented suppressive capacity of Treg cells. Likewise, with increasing amounts of cognate antigen the level of suppressive activity *in vitro* increases in parallel, suggesting that the strength of TCR signaling within Treg cells directly affects the magnitude of suppression [19]. On the other hand, Treg cells display broadly dampened activation of several signaling pathways upon TCR-mediated activation, for instance low phosphorylation of CD3ζ, SLP76, Erk1/2, and AKT [21–24]. Thus, altered kinase regulation in regulatory versus conventional T (Tconv) cells might explain the hyporesponsive state of Treg cells, characterized by impaired IL-2 expression and proliferation *in vitro* [23]. However, despite the reported differences between Treg and Tconv downstream of the TCR, it is still not fully understood how distinct components of the TCR signaling cascade influence Treg function.

The serine/threonine protein kinase C theta (PKCθ), which is predominantly expressed in T cells, plays an important role in signal transduction downstream of the TCR. T cells deficient in *PKCθ* show impaired NF-κB as well as NFAT and AP-1 activation, resulting in strongly decreased IL-2 expression and proliferation [25–27]. PKCθ is itself activated by DAG produced by phospholipase gamma 1, which is recruited to the TCR signaling complex via the LAT membrane after TCR engagement. PKCθ is the predominant PKC isotype that is rapidly recruited to the immunological synapse (IS) and is considered to negatively regulate the stability of the IS [28]. Results regarding the role of PKCθ in T cell differentiation and function, including the analysis of *PKCθ-*deficient mice in disease models, revealed the differential requirements of distinct T cell subpopulations for PKCθ. Several studies have shown that PKCθ is important in the regulation of Th2-mediated [29, 30] as well as Th17-dependent immune responses [31–36], but dispensable for Th1 responses [29, 37]. Interestingly, the subcellular location of PKCθ has been suggested to be different in Treg cells, in which PKCθ is excluded from the IS, but instead sequestered at the distal pole [38]. Furthermore, the same group has postulated that PKCθ, in contrast to its role in Tconv cells, provides a negative feedback on Treg function and, thus, Treg-mediated suppression is enhanced in the absence of PKCθ. In contrast, another study provided evidence that PKCθ is dispensable for Treg-mediated suppression [39]. Thus, the relation of PKCθ to Treg function has been somewhat controversial, which prompted us to re-evaluate its role in the activity of Treg cells. Using a combination of complementary genetic and pharmacological approaches specifically targeting the function of PKCθ, we could show that Treg cells exert their suppressive function independently of PKCθ.

## Materials and methods

### Mice

*PKCθ* knockout mice were as described previously [27]. All mouse lines were housed under specific pathogen-free conditions. The animal experiments were conducted in accordance with the Austrian Animal Welfare Law and Animal Experimental Act (BGBI No. 501/1988 and BGBI. No. 114/2012), and were approved by the Committee of the Animal Care of the Austrian Federal Ministry of Science and Research (BM:WFW-66.011/0064-WF/V/3b/2016).

### Thymocyte and splenocyte isolation, T cell sorting and *in vitro* CD4^+^ T cell activation

Single-cell suspensions of spleens and thymi were prepared by mechanical disintegration using metal sieves and cell strainers (Falcon), followed by the removal of erythrocytes by lyses (Mouse Erythrocyte Lysing Kit; R&D Systems). After a wash ing step with PBS/0.5% BSA/2 mM EDTA (viable) cell counts were determined with a LUNA Automated Cell Counter (Logos Biosystems). CD4^+^ T cells and naïve CD4^+^ T cells were sorted by MACS technology using a CD4^+^ T cell isolation or CD4^+^CD62L^+^ T cell isolation kit II, respectively, together with LS columns and a QuadroMACS Separator (all Miltenyi Biotec) according to the manufacturer’s instructions. The sort purity was checked by flow cytometry. T cell counts were adjusted to 2 x 10^6^/ml complete RPMI 1640 medium (supplemented with 10% heat-inactivated FCS; Biochrom), 2 mM L-Glutamine (Biochrom), 1% penicillin plus streptomycin (Biochrom), 10 μM 2-mercaptoethanol (Sigma), MEM nonessential amino acids (Sigma) and 1 mM sodium pyruvate (Sigma). For iTreg differentiation, naïve T cells were stimulated with plate-bound anti-CD3 (4 μg/ml, clone 2C11, produced in house) and anti-CD28 (1 μg/ml, clone 37.51; BD Biosciences) antibodies in the presence of recombinant TGF-β (5 ng/ml; eBiosciences) and human IL-2 (20 ng/ml; eBiosciences) and blocking anti-IL-4, anti-IL-12 and anti-IFNγ antibodies (all R&D). Cells were split 1:2 on day 3 of culture. For control siRNA experiments, CD4^+^ T cells were stimulated in complete RPMI with plate-bound anti-CD3 (5 μg/ml, clone 2C11, produced in house) and soluble anti-CD28 (1 μg/ml, clone 37.51; BD Biosciences). 2 days after transfection iTregs were used for *in vitro* suppression assay and Th0 cells were re-stimulated for 4 hours with plate-bound anti-CD3 (5 μg/ml) to address IL-2 mRNA expression by quantitative RT-PCR.

### *In vitro* suppression assay and AEB071 treatment

CD25^+^CD4^+^ and CD25^-^CD4^+^ T cells were isolated from erythrocyte-depleted cell suspensions of spleens and lymph nodes using the CD4^+^ T cell isolation kit II followed by CD25-PE and anti-PE MicroBeads (all Miltenyi Biotec) according to the manufacturer’s instructions. Sorted CD25^-^CD4^+^ T cells were labelled with 2.5 μM CFSE (Molecular Probes) for 4 min at 37°C; labelling was stopped by the addition of FCS. T cell-depleted splenocytes (using CD4 and CD8a MicroBeads; Miltenyi Biotec) treated for 45 min with 50 μg/ml mitomycin C (AppliChem) were used, after extensive washing, as antigen-presenting cells (APC). To induce proliferation, 0.5 μg/ml of anti-CD3 (clone 2C-11; BioLegend) was added. 1 x 10^5^ CFSE-labeled CD25^-^CD4^+^ responder T cells were cultured with 1 x 10^5^ APCs in 96-well U-bottom tissue culture plates (Falcon). CD25^+^CD4^+^ or CD25^-^CD4^+^ (non-Treg control) T cells were added at the ratios 1+1, 1+4 and 1+9. To address suppression by iTregs, *in vitro*-differentiated iTregs were harvested on day 5 of culture (see above) and dead cells were removed using a dead cell removal kit (Miltenyi Biotec) according to the manufacturer's instructions. For some experiments CD25^+^CD4^+^ and CD25^-^CD4^+^ T cells were treated with 1 μM AEB071, a potent and cell permeable low molecular weight inhibitor targeting the ATP binding site of protein kinase C isotypes (obtained from Novartis, Basel), for 30 min followed by 3 extensive washing steps with RPMI 1640 before addition to the co-culture assay. It has been shown previously that AEB071 markedly inhibits *in situ* PKCθ catalytic activity and effectively abrogates—at low nanomolar concentration early T cell activation, determined by IL-2 secretion and CD25 expression analyses [40]. On day 3 of co-culture, proliferation (based on CFSE-dilution) and activation (CD25 expression) was analyzed by flow cytometry; 7-AAD or propidium iodide was added to exclude dead cells from the analysis.

### siRNA transfection

Delivery of chemically synthesized short interfering RNA (siRNA) into CD4^+^ Tconv cells activated under non-polarizing conditions (“Th0”) or *in vitro* differentiated iTreg cells was accomplished using the Amaxa Nucleofector system and T cell Nucleofector Kits (Lonza) according to the manufacturer’s recommendations. On day 3 of culture, Th0 and iTreg cells were harvested (conditioned medium was collected) and transfected with 1.5 μM PKCθ or control siRNA (ON-TARGETplus mouse Prkc (18761) siRNA or scrambled; both Thermo Scientific) using program X-01. After transfection, performed in Nucleofector medium, conditioned medium (one third of the total volume) was added and the cells were cultured for an additional 2 days. On day 5 the silencing efficiency was analyzed by SDS Page/immunoblotting and/or intracellular staining/flow cytometry.

### Flow cytometry and luminex technology

Surface staining (including pre-incubation with FcR block; anti-CD16/32; BD Biosciences) was performed as described previously [41]. The following antibodies were used: anti-CD25 PE or APC, anti-CD4 FITC or APC, and anti-CD3 PECy7 (all from Biolegend). For the staining of intracellular antigens, the cells were fixed and subsequently permeabilized to the staining of surface antigens. The FoxP3 PE staining buffer set (eBiosciences) was used for the detection of Foxp3. For PKCθ fixation buffer and intracellular staining permeabilization wash buffer obtained from Biolegend and a mouse monoclonal anti-human PKCθ antibody (BD Biosciences) were used. Data were acquired on a FACSCalibur (CellQuest, BD Biosciences) and analyzed with FlowLogic software (eBioscience). The concentration of secreted IL-2 in the culture supernatants was measured by Luminex xMAP Technology using a Bio-Plex Pro Mouse Cytokine IL-2 Set (No. 171G5003M), according to the manufacturer´s instructions, on a Bio-Plex suspension array system (all Bio-Rad).

### RNA extraction, cDNA synthesis and real-time quantitative RT-PCR

Total RNA was isolated using the RNeasy Mini Kit (Qiagen) and reverse transcription was performed with the Omniscript Kit (Qiagen) and oligo-dT primers (Promega) according to the manufacturer's protocol. Gene expression was analyzed by quantitative real-time PCR using TaqMan technology on a 7500/7500 FAST Fast Real-Time PCR instrument (Applied Biosystems). The following reagents were used: 5x QPCR Mix (Rox) from Bio&SELL, TaqMan Gene Expression Assays mouse IL-2 (Mm00434256_m1) and Mouse GAPDH Endogenous Control (4352339E) (both Applied Biosystems). All amplifications were conducted in duplicates. The housekeeping gene GAPDH was used for normalization.

### SDS page and immunoblotting

Cell lysates from T cells were prepared and subjected together with precision plus protein standard (Bio-Rad) to SDS gel electrophoresis and immunoblotting as described previously [36]. Unspecific binding was blocked with 5% non-fat milk powder. Monoclonal antibodies detecting PKCθ (27/PKCθ) or Fyn (sc-16) were purchased from BD Biosciences and Santa Cruz Biotechnology, respectively. HRP-conjugated secondary antibodies against mouse and rabbit IgG were purchased from Thermo Scientific. For detection, SuperSignal West Pico Chemiluminescent Substrate (Thermo Scientific) was used and the bands were visualized using hyperfilm (Amersham Biosciences) and Superfix Enviro Safe 25 together with Roentrol Enviro Safe AC (Tental). Quantification was performed using ImageJ software.

### Statistical analysis

The data represent the mean (±SEM). FACS dot plots and immunoblots are shown as representative results (indicated in the figure legends). The number of mice used per experiment and the number of experiments performed as well as the statistical test used are listed in each figure legend. Experiments were repeated at least three times. The data were analyzed for statistical significance by the Mann-Whitney U test when comparing two groups, one sample t test when comparing data that are presented as relative values to the control group and Friedman test together with Dunn´s multiple comparison test when comparing multiple samples within one graph. These statistical analyses were performed with GraphPad Prism software (GraphPad Software Inc.). A p value ≤ 0.05 was considered statistically significant. Symbols used in the figures are: * p ≤ 0.05, ** p ≤ 0.01 and *** p ≤ 0.001.

## Results

### Treatment of CD25^+^CD4^+^ Treg cells with the PKC inhibitor AEB071 (Sotrastaurin) does not affect suppressive function

**Fig 1:**
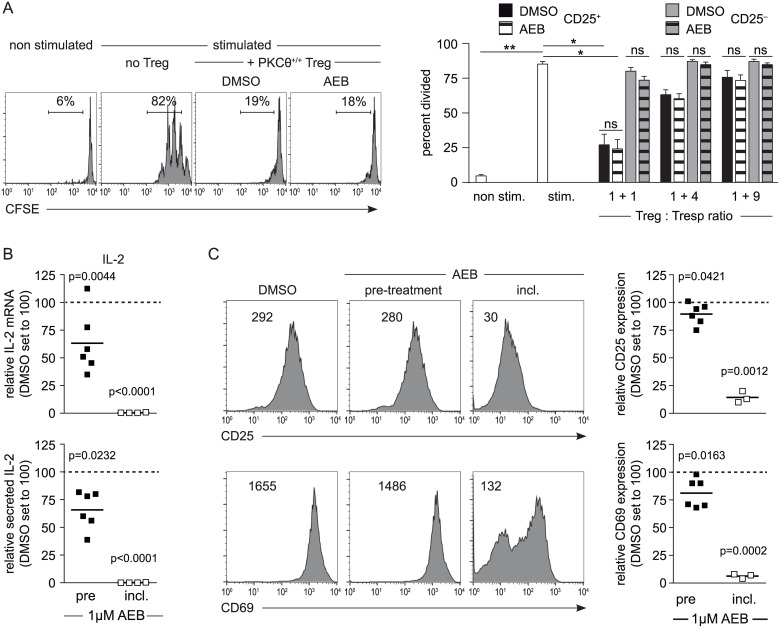
PKC inhibition by AEB071 does not affect the suppressive activity of Treg cells. (A) Wild-type CD25^-^ and CD25^+^CD4^+^ T cells were (pre-)treated for 30 min with 1 μM AEB071 or DMSO and added to the suppression assay after extensive washing. Representative histograms of CFSE staining (gated on CFSE^+^ CD4^+^ 7AAD^-^ cells) are depicted together with summarizing bar graphs of 3 independent experiments. No statistical significant difference (ns) was observed between DMSO- and AEB-treated wild-type Tregs when analyzed with the Friedman test together with Dunn´s multiple comparison test. (B, C) Wild-type CD4^+^ T cells were either (pre-)treated with DMSO or 1 μM AEB071 before the start of the culture or 1 μM AEB071 was added to the wells (incl.: AEB071 included during the whole period of culture). Activation was assessed by IL-2 expression (determined by Luminex technology and quantitative RT-PCR) as well as by CD25 and CD69 expression (analyzed by flow cytometry) on day 1. Representative FACS histograms of CD69 and CD25 (including the mean fluorescence intensity) are shown together with summarizing scatter dot blots. The data are presented relative to the DMSO control, which was set to 100 (dotted line). Each symbol represents results obtained with individual mice analyzed in at least 4 independent experiments. Statistical comparison to the DMSO control group was performed using one sample t test.

Based on the importance of TCR signaling for Treg biology and the critical role of PKCθ as a signaling molecule downstream of the TCR, we aimed to evaluate how the loss of PKCθ might affect Treg function. Previous studies have suggested that the pharmacological inhibition of PKCθ enhances the suppressive activity of Treg cells [38]. Thus, we analyzed whether the treatment of Treg cells with the highly selective PKC inhibitor Sotrastaurin (AEB071, [40]) affects their *in vitro* suppressive function. For this purpose, wild-type CD25^+^CD4^+^ Treg cells were incubated for 30 minutes with 1 μM AEB071 and washed extensively before being added to the *in vitro* suppression assay. This was to ensure that any AEB071 that would inhibit T responder cell activation was transferred along with the treated cells. These assays revealed that AEB071 pretreatment did not enhance or reduce Treg-mediated suppression of T cell proliferation (DMSO CD25^+^ versus AEB CD25^+^ in Fig 1A). CD25^-^ non-Treg cells, which were included in the assay as a control T cell subset, showed no suppressive capacity irrespective of the pre-treatment performed.

To validate whether the AEB071 used for the suppression assays was functional the inhibitor was included in the medium during the whole duration of the culture. Consistent with published results [40], inclusion of AEB071 strongly inhibited activation determined by CD25 and CD69 expression and almost completely abolished IL-2 expression (Fig 1B and 1C). In addition, we addressed if AEB071 pre-treatment (followed by several washing steps) before TCR stimulation does affect the activation of conventional CD4^+^ T cells. And indeed, 30 minutes pre-treatment with 1 μM AEB071 led to a reduced activation compared to the DMSO control. The strongest effect was observed for IL-2 expression but also visible analyzing the activation markers CD25 and CD69. This set of data proved that AEB071 was indeed functional at the concentration used; and also that pre-treatment, albeit less potent when compared to AEB071 inclusion, was sufficient to reduce TCR-induced activation of CD4^+^ T cells.

### PKCθ is dispensable for iTreg differentiation and function

**Fig 2:**
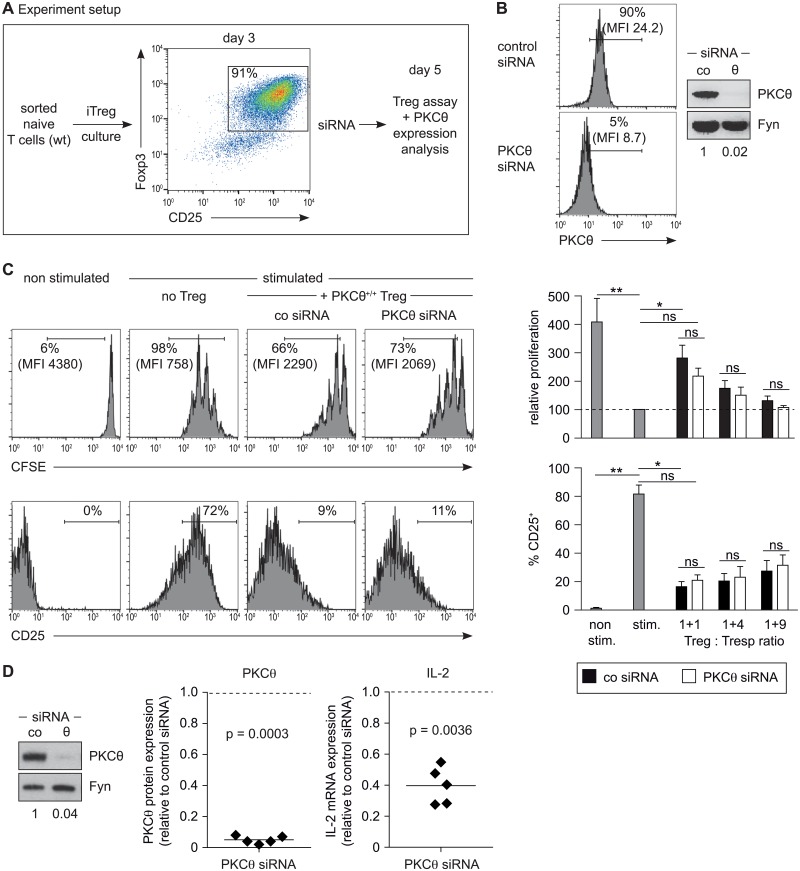
PKCθ knockdown by siRNA does not affect the suppressive function of iTreg cells. (A) Experimental setup: siRNA transfection of iTreg cells. (B) On day 3 of differentiation culture, iTreg cells were transfected with PKCθ or scrambled siRNA as a control (co) using the Amaxa transfection system. The silencing efficiency was analyzed 2 days after transfection by intracellular PKCθ staining and flow cytometry in addition by SDS Page and immunoblotting. The percentage of PKCθ-positive cells as well as the mean fluorescent intensity (MFI) of PKCθ staining is depicted in each histogram. Fyn was used as a loading control for the normalization and quantification of PKCθ protein expression (shown below the bands). (C) The suppressive capacity of iTreg cells transfected either with control or PKCθ siRNA was analyzed in co-cultures with CFSE-labeled CD25^-^CD4^+^ T cells (Tresp) stimulated with APCs and anti-CD3 antibodies. Histograms of CFSE and CD25 staining (gated on CFSE^+^ CD4^+^ 7AAD^-^ cells), including values of percentage divided and mean fluorescence intensity (MFI), and bar graphs summarizing results of 4 independent experiments are shown. Proliferation is depicted relative to stimulated Teff in absence of Tregs, whose mean CFSE fluorescence intensity was set to 100 (dotted line). No statistical significant difference (ns) was observed between control and PKCθ-siRNA transfected Tregs with the Friedman test together with Dunn´s multiple comparison test. (D) Knockdown of PKCθ in CD4^+^ Tconv cells (transfected on day3 of culture) was analyzed by SDS Page and immunoblotting 2 days after transfection. The silencing efficiency is depicted below the bands and in the summarizing scatter dot plot. To determine the IL-2 expression transfected CD4^+^ T cells were re-stimulated 2 days after transfection for 4 hours with anti-CD3 antibodies and IL-2 mRNA was determined by quantitative RT-PCR. Expression was normalized to the house keeping gene GAPDH and represented as fold of control siRNA. Dotted line: expression of control siRNA treated cells set to 1. Each symbol represents results obtained with cells isolated from individual mice. Statistical comparison to the control siRNA group was performed using one sample t test. ns = not significant.

In addition to pharmacological inhibition we applied siRNA transfection to specifically silence PKCθ. These experiments were performed with wild-type iTregs already differentiated *in vitro* in the presence of IL-2 and TGF-β for three days, which, in contrast to *ex vivo* CD25^+^CD4^+^ Treg cells, showed a good PKCθ knockdown efficiency with acceptable survival rates. The silencing of PKCθ expression by siRNA was analyzed two days after siRNA transfection (on day five of iTreg culture) by SDS Page/immunoblotting and intracellular staining/flow (the experimental setup is depicted in Fig 2A). These experiments demonstrated a strong reduction of PKCθ expression on the protein level (Fig 2B). In accordance with our results obtained with AEB071, we observed a comparable suppression of T responder cell activation (CD25 expression and IL-2 secretion) and proliferation (CFSE dilution) by iTreg cells transfected either with control or PKCθ siRNA (Fig 2C and not shown). In contrast, silencing of PKCθ by siRNA in conventional CD4^+^ T cells at day 3 of culture affected their activation leading to decreased IL-2 secretion analyzed 2 days after siRNA transfection (Fig 2D). Consistently, AEB071 pretreatment of wild-type iTreg—similar to the *ex vivo* CD25^+^ Treg cells shown in Fig 1 - did not affect the suppressive activity at all (data not shown).

**Fig 3:**
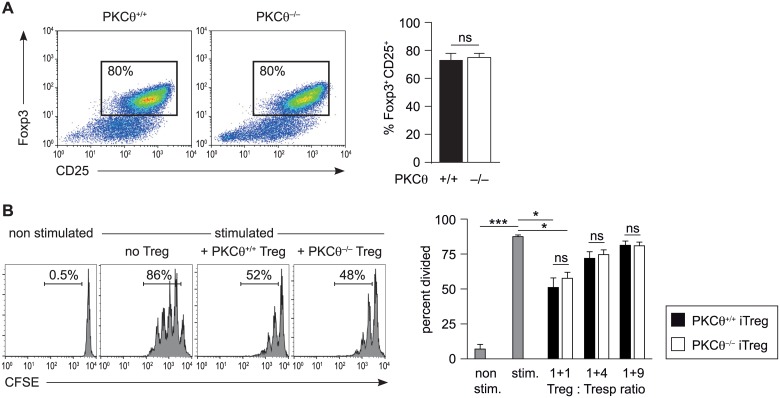
PKCθ is dispensable for iTreg cell differentiation and function. (A) Naïve CD4^+^ T cells isolated from PKCθ^+/+^ and PKCθ^-/-^ mice were differentiated *in vitro* under iTreg-inducing conditions (IL-2/TGF-β) and analyzed for Foxp3 and CD25 expression by flow cytometry on day 5 of culture. Representative FACS dot plots of Foxp3 and CD25 staining together with summarizing bar graphs are shown (n = 8). Statistical significance was determined using the Mann-Whitney U test. (B) The suppressive capacity of PKCθ^+/+^ and PKCθ^-/-^ iTreg cells was analyzed in co-cultures with CFSE-labeled CD25^-^CD4^+^ T cells (Tresp) stimulated with APCs and anti-CD3 antibodies. Representative histograms of CFSE staining (gated on CFSE^+^ CD4^+^ 7AAD^-^ cells) are depicted together with summarizing bar graphs of 4 independent experiments. Statistical analysis was determined using Friedman test together with Dunn´s multiple comparison test. ns = not significant.

Furthermore, PKCθ has been implicated as a negative regulator in iTreg development [42]. This finding prompted us to re-evaluate whether PKCθ deficiency affects the *in vitro* differentiation of naïve CD4^+^ T cells towards iTreg cells, induced by IL-2 and TGF-β. PKC*θ*^+/+^ and PKCθ^-/-^ iTreg cells showed a similar expression of Foxp3 and CD25 when analyzed on day 5 of differentiation by flow cytometry (Fig 3A). Also, the suppressive capacity of these *in vitro*-generated iTreg cells was comparable between both genotypes (Fig 3B). Together, these results suggest that while PKCθ is essential and non-redundant in Tconv cell activation, it is dispensable for the generation and function of iTreg cells, at least *in vitro*.

### *PKCθ*-deficient mice have reduced but functional CD25^+^Foxp3^+^ CD4^+^ Treg cells

**Fig 4:**
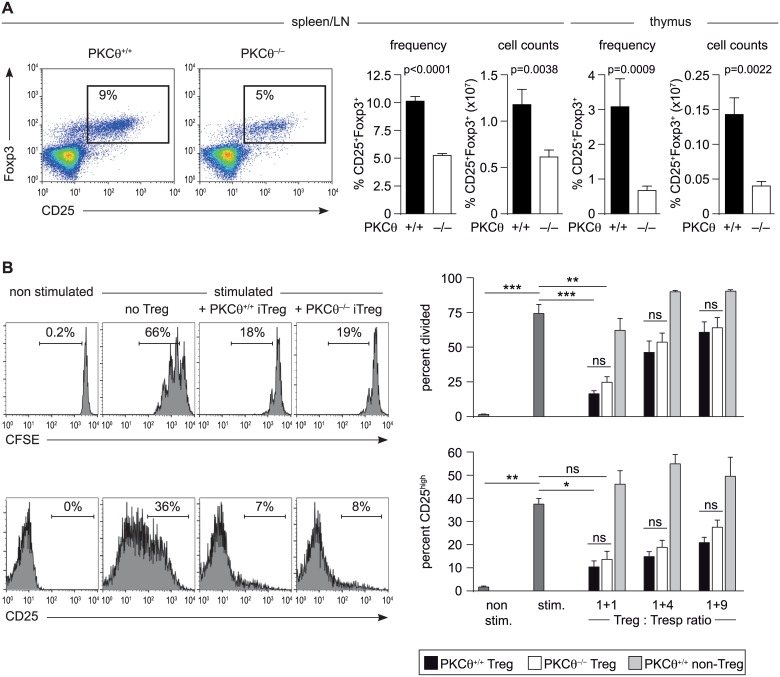
PKCθ deficiency impairs Treg development but does not affect suppressive activity. (A) The frequency and cell counts of Treg cells in the spleen/lymph node (LN) and thymus of PKCθ^-/-^ and PKCθ^+/+^ mice was analyzed by flow cytometry. Representative FACS dot plots of Foxp3 and CD25 staining (spleen/LN cells, gated on CD3^+^CD4^+^) together with summarizing bar graphs are shown (n ≥ 11 for spleen/LN, n ≥ 6 for thymus). Statistical significance was determined using the Mann-Whitney U test. (B) The suppressive capacity of PKCθ^+/+^ and PKCθ^-/-^ CD25^+^CD4^+^ T cells (Treg) was analyzed in co-cultures with CFSE-labeled CD25^-^CD4^+^ T cells (Tresp) stimulated with APCs and anti-CD3 antibodies. Representative flow cytometric dot blots of CFSE and CD25 staining (gated on CFSE^+^ CD4^+^ 7AAD^-^ cells) are depicted together with summarizing bar graphs of 3 independent experiments (duplicates per experiment). Wild-type CD25^-^CD4^+^ T cells (non-Treg) were included as controls. Statistical analysis was determined using Friedman test together with Dunn´s multiple comparison test. ns = not significant.

Additionally, we addressed the role of PKCθ in CD25^+^CD4^+^ Treg cell development *in vivo* by comparing *PKCθ*-deficient and wild-type mice. In line with previous observations, flow cytometric analyses revealed significantly reduced frequencies of Foxp3^+^CD25^+^ CD4^+^ T cells in the thymus and secondary lymphoid organs of mice lacking PKCϴ (Fig 4A). Importantly, however, PKCθ^+/+^ and PKCθ^-/-^ CD25^+^CD4^+^ Treg cells showed comparable suppressive capacities in the *in vitro* suppression assay: CD25^+^CD4^+^ T cells isolated from *PKCθ*-deficient mice were able to suppress the activation (CD25 expression) and proliferation (CFSE dilution) of wild-type CD4^+^ T cells to the same degree as CD25^+^CD4^+^ T cells from wild-type mice (Fig 4B). Moreover, equal suppressive activity of Treg cells from both genotypes was observed using *PKCθ*-deficient CD4^+^ T responder cells (data not shown), although the overall activation and proliferation of PKCθ^-/-^ T responder cells was reduced. Furthermore, no difference in the expression of several Treg signature genes (*ctla4*, *gpr83*, *ikzf4*, *il2rb*, *il10*, *tnfrsf18*, *foxp3*, *il2ra*) analyzed on mRNA level in sorted CD25^+^CD4^+^ from wild-type and *PKCθ*-deficient mice was observed (data not shown). These results suggest that PKCθ is important for the development of thymus-derived Foxp3^+^CD25^+^ CD4^+^ T cells, but dispensable for Treg-mediated suppression of T cell activation.

## Discussion

The activation of Treg cells via the TCR has been suggested to be mandatory for their suppressive function [12–14, 17–20]. One interesting player in this regard is the serine/threonine kinase PKCθ, which acts downstream of the TCR in Tconv cells and is crucial for their activation and IL-2 expression upon TCR engagement [25–27]. Nevertheless, in contrast to its well-established role in Tconv activation, the requirements for PKCθ in Treg-mediated suppression are much less apparent. Here, we demonstrated that the ability of Treg cells to suppress Tconv activation and proliferation is independent of functional PKCθ. Our results obtained with *PKCθ*-deficient mice are in accordance with the study by Gupta *et al*. showing that PKCθ^-/-^ Treg cells are as potent as PKCθ^+/+^ Treg cells in the inhibition of Tconv [39]. However, since knockout in mice may be limited by genetic compensation and a redundancy of functions, we applied pharmacological inhibition of PKC by the highly selective low-molecular-weight immunosuppressant (Sotrastaurin, [40]) as well as silencing by PKCθ isotype-specific siRNA to circumvent this problem. Using both these approaches we observed, in agreement with the results obtained with *PKCθ*-deficient mice, that PKCθ is dispensable for the suppressive function of Treg cells. Nonetheless, it has been shown that the novel and conventional PKC isotypes PKCθ and PKCα have partially redundant roles in alloimmune responses and converge to regulate IL-2 [43]. Furthermore, PKCθ and PKCα have been described as the main PKC isotypes involved in the downregulation of engaged and non-engaged TCR complexes on the surface of T cells, respectively [44]. Thus, PKCθ and PKCα seem to have a certain degree of overlapping functions in T cell responses. However, using the siRNA approach we specifically silenced PKCθ and thereby focused on its role in Treg-mediated suppression, for which PKCθ proved to be fully dispensable, as already mentioned. Interestingly, another novel PKC isotype, PKCη, has been implicated as a positive regulator in suppression by Treg cells [45]. PKCη interacts with CTLA-4 and localizes at the IS in Treg cells after stimulation, while in Tconv cells PKCθ and CD28 co-localize at the IS [46]. Of note, one hallmark of Treg cells is a high expression of CTLA-4, which displaces CD28 from the IS [47], generating a Treg IS distinct of Tconv IS.

In line with these findings, it was shown by a different group that PKCθ is excluded from the Treg IS and, furthermore, that PKCθ acts as an inhibitor of Treg-mediated suppression [38]. The authors demonstrated that pretreatment of Treg cells for 30 minutes with the PKCθ inhibitor C20 is sufficient to enhance the suppressive activity, which is in strict contrast to our findings using panPKC-selective AEB071 pretreatment for the same duration. Of note, although pre-treatment of Tconv cells for 30 minute at a concentration of 1 μM, was sufficient to inhibit Tconv activation to a certain degree; this treatment regime did not affect Treg-mediated suppression. Even the very high concentration of AEB071 (5 μM) applied for 90 minutes did not at all impact on suppression by Tregs analyzed in co-culture settings (unpublished observations). However, AEB071 was fully able to suppress Tconv activation when included in the cultures throughout, implying a reversibel nature of AEB071. Consistent with our observations, in a different study that analyzed the effect of PKCθ inhibition on Treg function, the suppressive activity of human Treg cells was not affected by AEB071 or C20 included in the cultures for one week before the suppression assay was performed [48].

Stimulation via the TCR is not only critical for suppressive activity, but is also of great importance for Treg development in the thymus. In particular, increased affinity and TCR signaling strength are required for the initiation of a Treg differentiation program and the induction of Foxp3 expression [8, 9, 49]. Interestingly, in contrast to its dispensable role in Treg function, PKCθ seems to play an important role in the thymic generation of Treg cells. This is illustrated by a decreased number of Foxp3^+^CD25^+^ CD4^+^ T cells in the thymus and secondary lymphoid organs of *PKCθ* knockout mice, which is consistent with a previous publication [39]. Mechanistically, it has been suggested that PKCθ stimulates Foxp3 promoter activity in a NFAT-dependent manner. However, since also/additionally IL-2 is essential for Treg differentiation [50–53] and since PKCθ is a known positive regulator of IL-2 expression [25–27], the possibility remains that the reduced IL-2 level in *PKCθ*-deficient mice is responsible for the impaired Treg development. Of note, mice lacking the PKC isotypes PKCαα and PKCη show normal Treg numbers [45, 54], suggesting an isotype-specific requirement of PKCθ in thymic Treg development. In contrast to its role in the generation of thymus-derived Treg cells, we did not observe any role of PKCθ in iTreg differentiation induced *in vitro*, which is in contrast to the work of Ma *et al*. [42], who observed that PKC inhibits Foxp3 expression and, thus, iTreg differentiation. However, iTreg differentiation cultures depend on the addition of TGFβ and high amounts of IL-2 for efficient iTreg induction. Therefore, we cannot exclude that exogenous IL-2 overrides the need for PKCθ. Taken together, our findings demonstrate that while Tconv cell activation strictly depends on PKCθ, Treg cells do not require this PKC isotype to exert their suppressive function. This is of importance with regard to the ability to specifically target effector T cells with a PKCθ-selective small-molecule drug without interfering with Treg function.
